# Removal of dead fish eggs by *Asellus aquaticus* as a potential biological control in aquaculture

**DOI:** 10.1038/s41598-024-57903-4

**Published:** 2024-03-27

**Authors:** Balázs Kucska, Quyến Nguyễn Ngọc, Bence Ivánovics, Ádám Staszny, Máté Havasi, Károly Vranovics, Jeffrey Daniel Griffitts, Ádám Varga, Béla Urbányi, Tamás Müller

**Affiliations:** 1 Institute of Aquaculture and Environmental Safety, Department of Freshwater Fish Ecology, Hungarian University of Agriculture and Life Sciences, Páter Károly U. 1., Gödöllő, 2100 Hungary; 2 Institute of Aquaculture and Environmental Safety, Department of Environmental Toxicology, Hungarian University of Agriculture and Life Sciences, Páter Károly U. 1., Gödöllő, 2100 Hungary; 3 Institute of Aquaculture and Environmental Safety, Department of Applied Fish Biology, Hungarian University of Agriculture and Life Sciences, 8360 Keszthely, Hungary; 4 Institute of Aquaculture and Environmental Safety, Department of Aquaculture, Hungarian University of Agriculture and Life Sciences, Páter Károly U. 1., Gödöllő, 2100 Hungary

**Keywords:** Freshwater ecology, Agroecology, Hydrology, Ichthyology

## Abstract

The objective of the present experimental study was to gain a better understanding of the foraging activity of *Asellus aquaticus* during fish egg incubation. *A. aquaticus* were introduced into experimental setups of dead eggs, viable eggs and hatched larvae of zebrafish (*Danio rerio*), a commonly used model organism. The amount of *A. aquaticus* and the duration of their exposure to the eggs significantly affected the proportion of consumed dead eggs in each experimental cycle. *A. aquaticus* belongs to the group of aquatic detritivores, and no predatory behavior was observed during the experiments. These crustaceans could distinguish between the dead eggs and those containing living embryos. Furthermore, zebrafish larvae remained unharmed by *A. aquaticus*, even in the absence of alternative food source. These findings underscore the potential sanitary role of these crustaceans in natural waters and offer new perspectives on their possible use as biological control organisms in aquaculture hatcheries. Additionally, our results suggest a potential application of *A. aquaticus* in combating pathogens by reducing the growth substrates for bacteria and fungi.

## Introduction

The hatching success of fish eggs depends on many factors in nature and in commercial fish producing systems as well. Among many factors, genetic compatibilities, maternal and ova characteristics, spermatozoa motility and sperm/egg ratio play a significant role in fertilization success and the hatching rate of fish embryos^1–3^. Besides the internal biological features, environmental factors such as optimal temperature, salinity, predators, chemical pollutants, and diseases are all crucial factors for successful hatching^4–7^.

Bacterial and fungal infections are a primary concern during the incubation period of fish eggs, especially in aquaculture hatcheries^8–11^. Harmful pathogens such as *Saprolegnia* sp., *Flavobacterium* sp. or *Pseudomonas* sp. are commonly and naturally found in many water systems. The mucous layer of eggs appears to be a good substrate for adhesion and colonization by many of these pathogens^12^.

It is generally observed that the appearance of pathogens, such as bacteria and fungi, correlates with the proportion of dead eggs^4,13^. However, it is possible that increased growth of bacteria and fungi is a result of, not necessarily a cause of, increased mortality. On the other hand, dead eggs may promote the spread of horizontally transmitted diseases. Prior studies have shown *Saprolegnia parasitica* to initially colonize dead eggs and the hyphae from these then infect the surrounding living embryos^14^. Smith et al.^13^ showed that only dead eggs are infected by zoospores, whereas both live and dead eggs were infected by hypheal growth from adjacent infected eggs. In contrast, it has also been observed that *S. diclina* from fungus-infected salmonids can directly infect live eggs without any need for prior colonization of dead eggs^15^.

In addition to the potential transmission of diseases between eggs, there could be other adverse effects resulting from a high quantity of unfertilized and deceased eggs within a water system. Dead and ruptured eggs may provide considerable nutrients for heterotrophic bacteria by leaching^16^. Large numbers of bacteria can have high oxygen requirements^17^, producing metabolic by-products^18^ or toxins^19^. The proliferation of heterotrophic bacteria not only influences egg survival rate but it has negative impacts on a recirculating aquaculture system (RAS) via the competition with nitrifying bacteria^20^.

The physical barrier of the chorion and membranes defends against invading pathogens^21^; after hatching, the exposure to pathogens dramatically increases. Colonized dead eggs also create a route for pathogen transmission towards the freshly hatched larvae.

Various techniques such as egg disinfection have been applied to control egg pathogens^8,10^ in commercial aquaculture systems. Chemical treatments have been used to control disease transmission with varying levels of success. However, the elimination of dead eggs, with their potential as a substrate for disease and infection in fish, has been of limited focus. In a separate study, the mechanical removal of deceased eggs of Chinook salmon (*Oncorhynchus tshawytscha*) at the eyed stage resulted in a reduction in bacterial counts^4^. This technique could be applicable to salmonids owing to their relatively larger egg size, enhanced visibility, and the absence of a swirling incubation medium. However, it entails a significant investment of time and labour. For other species (e.g., cyprinids or percids) in which egg size is considerably smaller or incubation occurs in turbulent media or on surfaces^22^, the manual elimination of dead eggs becomes challenging, ineffective, and at times unfeasible.

Thus, biological control methods can serve as effective alternatives to chemical or labour-demanding mechanical disease management. The concept of the biological control of pests is gaining interest as the harmful effects of agrochemicals continues to be understood. Grass carp, *Ctenopharyngodon idella,* is one of the best-known examples of a biological control “tool” to manage invasive aquatic plants^23^. Several species of wrasse (*Labridae*) are used as cleaner fish to remove salmon lice (*Lepeophtheirus salmonis*) from farmed Atlantic salmon (*Salmo salar*)^24^. The first application of macroinvertebrates as a biological control of fungal disease was reported by Oseid^25^. In this study, it was shown that grazing on the mycelia of oomycetes infected eggs by *Asellus militaris* and *Gammarus pseudolimnaeus* increased the hatching rate of walleye embryos (*Sander vitreum*). However, the application of crustaceans as a biological control in fish hatcheries is still not very common.

*A. aquaticus* is a native detritivore abundant in most European freshwater ecosystems. High densities can be found in waters with organic pollution or in relatively clean water with high levels of naturally occurring organic matter^26^. Several studies have concluded that *A. aquaticus* selectively feed on detritus colonized by microorganisms, in particular fungi^27,28^. We do not have proof that fish eggs which are not infected by pathogenic fungi attract *A. aquaticus*. Our hypothesis was that *A. aquaticus* can consume not just the mycelia from eggs infected with oomycetes, but is also capable of eliminating or reducing the number of dead eggs before the excessive outgrowth and spread of pathogenic fungi, without harming living embryos or freshly hatched larvae. We implemented three experimental cycles, investigating the foraging preferences of *A. aquaticus* using different ratios of living and dead eggs/embryos and freshly hatched larvae of zebrafish (*Danio rerio*) and evaluating the effects of *A. aquaticus*’ presence on larval vitality.

## Materials and methods

### Ethics statement

According to the European Directive on the protection of animals used for scientific purposes (2010/63/EU) zebrafish embryos/larvae are not subject to ethical regulations until they reach independent feeding stage (120 hpf)^29^. No independently feeding zebrafish larvae (older than 120 hpf) were involved in our experiments.

### Zebrafish maintenance

A genetically unmodified, laboratory zebrafish strain (AB) was used for the experiments. This line has been bred in the Zebrafish Laboratory of the Hungarian University of Agriculture and Life Sciences (HUALS). Fish were maintained in 3 L polycarbonate tanks in a recirculated system (ZebTEC, Tecniplast S.p.a., Italy) through an upwelling bead filter at 25 ± 2 °C, and fed three times per day with commercial flakes (Sparos Zebrafeed, 400–600 µm) and live *Artemia nauplii* grown from cyst (Ocean Nutrition > 230000NPG). The photoperiod was set to 14 h light: 10 h dark. Guidelines from the good laboratory practice (GLP—Organization for Economic Co-operation and Development (OECD)) and Institutional Animal Care and Use Committees of the Hungarian University of Agriculture and Life Sciences were followed for animal care. The National Food Chain Safety Office, Animal Health and Animal Welfare Directorate of Government Office of Pest County has registered the site of the Zebrafish Laboratory as an animal testing user, breeder, and supplier facility and authorized its operation under the file number PEI/001/1719–2/2015 (Appendix I.).

### Broodstock of *A. aquaticus*

*A. aquaticus* specimens were collected from an experimental recirculating aquaculture system (RAS) of HUALS designed for fish broodstock housing. Three 5000 L fish tanks were connected and operated as a freshwater RAS which included a drum filter, moving bed bio reactor and aeration. Daily water exchange varied between 2 and 6% of the total volume depending on the biomass kept in the tanks. The system has been in operation for over five years without shut down. For broodstock we offered live zooplankton as supplementation. We assume that *A. aquaticus* appeared in the system owing to this practice as zooplankton was collected from natural waters. The starting population then multiplied on the organic particles in the effluent water. *A. aquaticus* was collected from the effluent water with a plastic sieve in a weekly quantity of approximately 200–1500 individuals. Specimens for the experiments were chosen randomly, the only criteria were that adult individuals (4–5 mm) were used.

### Propagation, egg collection and incubation prior to the experiment

During propagation, five zebrafish females with five males were introduced into each breeding tank (n = 6). According to the protocol of zebrafish fertilization and embryo isolation (http://www.zfic.org/common%20techniques/mating.pdf), all fish were released to spawn. 1.7 L breeding tanks (ZebTEC, Tecniplast S.p.a., Italy) were used in all experiments. Water conditions were: temperature 25 °C; pH 7.0 ± 0.2; average conductivity 525 µS. Eggs were collected from each tank and placed into Petri dishes (Ø 100 mm) 2 h after the onset of light. Eggs were incubated in an incubator (25.5 °C, photoperiod was set at 14 h light: 10 h dark) with daily water changes. After a 24 h incubation period, all eggs were checked and separated into two batches: dead eggs (white) and living eggs (transparent with living embryos). The number of dead eggs represents both unfertilized eggs and embryos that have died during the first 24 h of embryogenesis.

### Incubation of zebrafish embryos in the presence of *A. aquaticus*: experimental design

The experiments were carried out in sterile, flat-bottom, non-treated, multiwell-plates with a lid (6—well format, Vtotal = 5 mL, Vwater = 4 mL). In the first experiment (E1), *A. aquaticus* individuals (total body length 4–5 mm) were added to some of the wells in varying numbers (1, 3 or 5 per well; Table 1). Prior to the experiments, *A. aquaticus* specimens were starved in each well for 24 h. Varying ratios of dead eggs and living embryos were introduced into the wells at 24 h post-fertilization (hpf) (Table 1). Each well contained a piece of biological filtration media (AQ-09KL 436 m^2^/m^3^, Ø/ = 9/7 mm, Aquacultur GmBH), which served as a hiding/hanging place for *A. aquaticus*. In order to provide identical experimental conditions, the control wells without *A. aquaticus* contained the same media. The number of living embryos and dead eggs were checked every five hours till the end of E1. In the second experiment (E2), dead eggs, freshly hatched larvae (non-feeding stage) and *A. aquaticus* (total body length 4–5 mm) were introduced into the wells at different ratios (see Table 1). The number of larvae and dead eggs was monitored every ten hours until the end of the experiment. Water temperatures were 27.4–27.8 °C and 24.5–24.7 °C during E1 and E2, respectively. The photoperiod was set at 14 h light: 10 h dark without water changes during the experimental cycles.

**Table 1:** Summary of the experimental design for each experimental series.

	1 well contains			No of repetitions/treatment (n =)	Duration (h)
	No of living embryo^#^ or larvae^##^ (n =)	No of dead eggs (n =)	No of *A. aquaticus* (n =)		
Experiment 1 (E1)^**#**^	20	10	Ø	12	45
			1	12	
			3	12	
			5	12	
Experiment 2 (E2)^**##**^	5	5	Ø	12	40
	5	5	1	12	
	5	5	3	12	
	5	5	5	12	
	5	Ø	Ø	12	
	5	Ø	1	12	
	5	Ø	3	12	
	5	Ø	5	12	
Experiment 3 (E3)^**#**^	5	Ø	Ø	6	96
	5	3	Ø	6	
	5	Ø	3	6	
	5	3	3	6	

The number of dead eggs represents both unfertilized eggs and embryos that have died during the first 24 h of embryogenesis.

### Evaluation of larval vitality

To evaluate the effects of *A. aquaticus* presence on the vitality of zebrafish larvae, experiment 3 (E3) was performed. Normally developed 24 hpf zebrafish embryos were placed in 6-well cell culture plates (5 embryos/well) containing 10 mL system water per well and incubated at 25.5 ± 0.5 °C until 118 hpf in the presence or absence of *A. aquaticus* (3 individuals/well) and in the presence or absence of dead zebrafish eggs (3 dead eggs/well) (n = 30 larvae/group). We provided one piece of biofilter media per well as shelter for *A. aquaticus*. The experimental groups were as following: (I) control (living embryos); (II) living embryos + dead eggs; (III) living embryos + *A. aquaticus*; (IV) living embryos + dead eggs + *A. aquaticus* (Table 1). Mortality was monitored daily. At 118 hpf, *A. aquaticus* individuals and biofilter media were carefully removed. At the end of the experiment (E3) morphological and behavioral alterations were detected (see below).

### Detection of morphological alterations (E3)

Zebrafish larvae were anesthetized, positioned laterally and imaged under a stereo microscope (Leica M205 FA, Leica DFC7000T camera, Leica Application Suite X software, Leica Microsystems GmbH, Germany). The standard and total body length, the swim bladder area, and the length of the gut were assessed using ImageJ software (n = 30 larvae/group). The approximate gut length was determined based on the work of Chuang et al.^30^.

### Zebrafish behavioral assay (E3)

Zebrafish larvae were individually transferred into 96-well cell culture plates containing 200 µL system water per well (n = 30 larvae/group). Next, the 96-well plates were placed (one by one) in a ViewPoint Behavior Technology instrument and the larvae were monitored during a 10 min light—20 min dark—10 min light incubation period. The ViewPoint ZebraLab system continuously tracked the movement of the larvae and analyzed their locomotor activity (e.g., the total distance covered by the larvae in small or in large movements) at one-minute intervals. To evaluate alterations in the locomotor activity of the larvae, we segmented the behavior test into distinct periods and determined the total distance traveled by the larvae in one minute during each specified period. These periods included: the pre-transition period (5 min before the light–dark transition), light–dark transition (the first one minute of the dark phase) and first and second halves of the dark phase.

### Statistical analysis

The effects of time and the number of *A. aquaticus* was tested in the first experiment (E1) using a Multivariate Analysis of Variance (MANOVA), in which the ’number of eggs containing living embryos’, the ’number of dead eggs’ and the ’number of hatched larvae’ was considered as dependent variables and ’hours’ and the ’number of *A. aquaticus*’ were factors. In the second (E2) experiment, there was no variation in the number of larvae, therefore a two-way factorial ANOVA was performed, in which ’number of dead eggs’ was considered as the dependent variable, and ’hours’ and the ’number of *A. aquaticus*’ were factors. Dependent variables were transformed using log(x + 1) to satisfy the assumptions of MANOVA and ANOVA. All analysis were conducted in SPSS v25 statistical software. Data derived from zebrafish vitality tests were analyzed by one-way parametric ANOVA and post-hoc Tukey test or Kruskall-Wallis test and post-hoc Dunn test using the GraphPad Prism 8 software. Results are presented as the mean ± standard deviation (SD).

## Results

### Effects of *A. aquaticus* presence on the number of dead eggs, living embryos and hatched larvae (E1)

**Figure 1:**
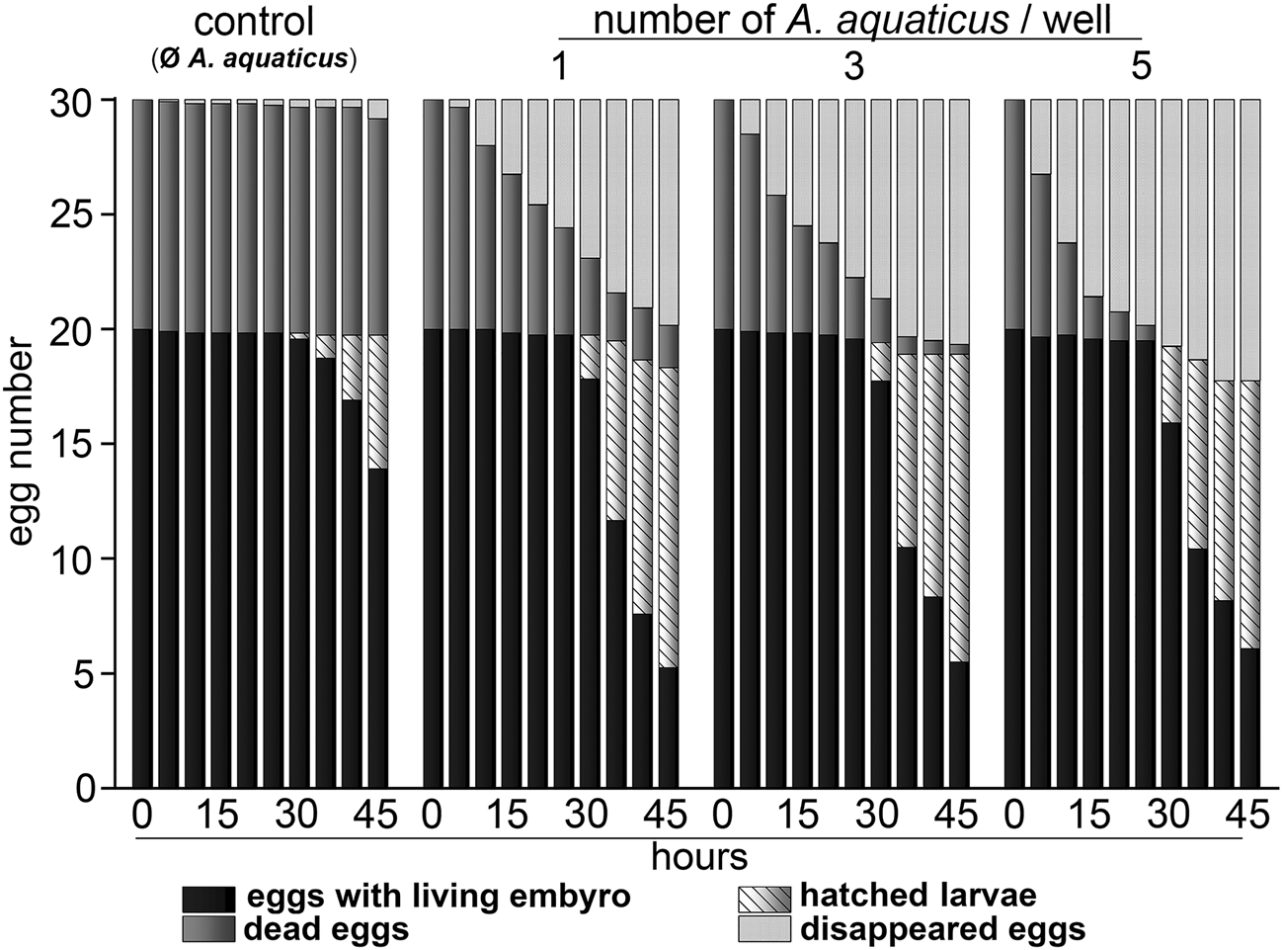
Effects of *A. aquaticus* on the number of dead eggs, living embryos and hatching rate during the incubation period (E1). Stacked bar chart presents the average number of dead eggs, living embryos, disappeared eggs and hatched larvae in presence or absence of *A. aquaticus* over a 45-h incubation period. Initially, each replicate was introduced with 10 dead and 20 living embryos. The bar graphs indicate that a substantial number of the introduced dead eggs disappeared during incubation when *A. aquaticus* was present.

In the first experiment, the number of dead eggs continuously decreased after the first 5 h in *A. aquaticus* containing wells. The decreasing proportion of dead eggs accelerated according to the number of *A. aquaticus* specimens/well. At the first assessment time (5:00 h), the number of eggs were statistically different (p < 0.05) between the experimental groups and this difference increased with time (Tables 2 and 3, Fig. 1). The first larva hatched during the 30th hour in every experimental group, however, the hatching process was more dynamic in the presence of *A. aquaticus* compared to control.

**Table 2:** Summarised data of Experiment 1, at the 45th hour.

	Eggs with living embryo (%)	Dead eggs (%)	Hatched larvae (%)
Control	69.6 ± 18.9^a^	94.2 ± 7.9^a^	29.2 ± 18.2^a^
1 *A. aquaticus*/well	26.3 ± 29.2^b^	18.3 ± 15.9^b^	65.4 ± 25.4^b^
3 *A. aquaticus*/well	27.5 ± 16.3^b^	4.2 ± 6.7^c^	67.1 ± 14.2^b^
5 *A. aquaticus*/well	30.4 ± 26.2^b^	0	58.3 ± 22.1^b^

Values for fertilised eggs and hatched larvae are expressed in the percentage of the initial number of viable eggs. Values for dead eggs are expressed in the percentage of the initial number. Data are presented as means and standard deviations (± SD). Different superscripts indicate statistical differences.

**Table 3:** Summarised statistical analysis (MANOVA) of the effects of time, the number of *A. aquaticus* and their combination, on egg batches in Experiment 1.

		df	Mean squares	F	p
Hours	FECLE	9	2.324	26.066	< 0.001
	UF	9	5.527	48.34	< 0.001
	Hatched larvae	9	16.148	221.206	< 0.001
*A. aquaticus*	FECLE	2	1.41	15.806	< 0.001
	UF	2	50.474	441.453	< 0.001
	Hatched larvae	2	3.293	45.112	< 0.001
Hours × *A. aquaticus*	FECLE	18	0.393	4.403	< 0.001
	UF	18	2.054	17.961	< 0.001
	Hatched larvae	18	0.67	9.172	< 0.001
Error	FECLE	330	0.089		
	UF	330	0.114		
	Hatched larvae	330	0.073		

*FECLE* fertilised eggs containing living embryos, *UF* unfertilised/dead eggs.

### Effects of *A. aquaticus* presence on the number of dead eggs and living larvae (E2)

**Figure 2:**
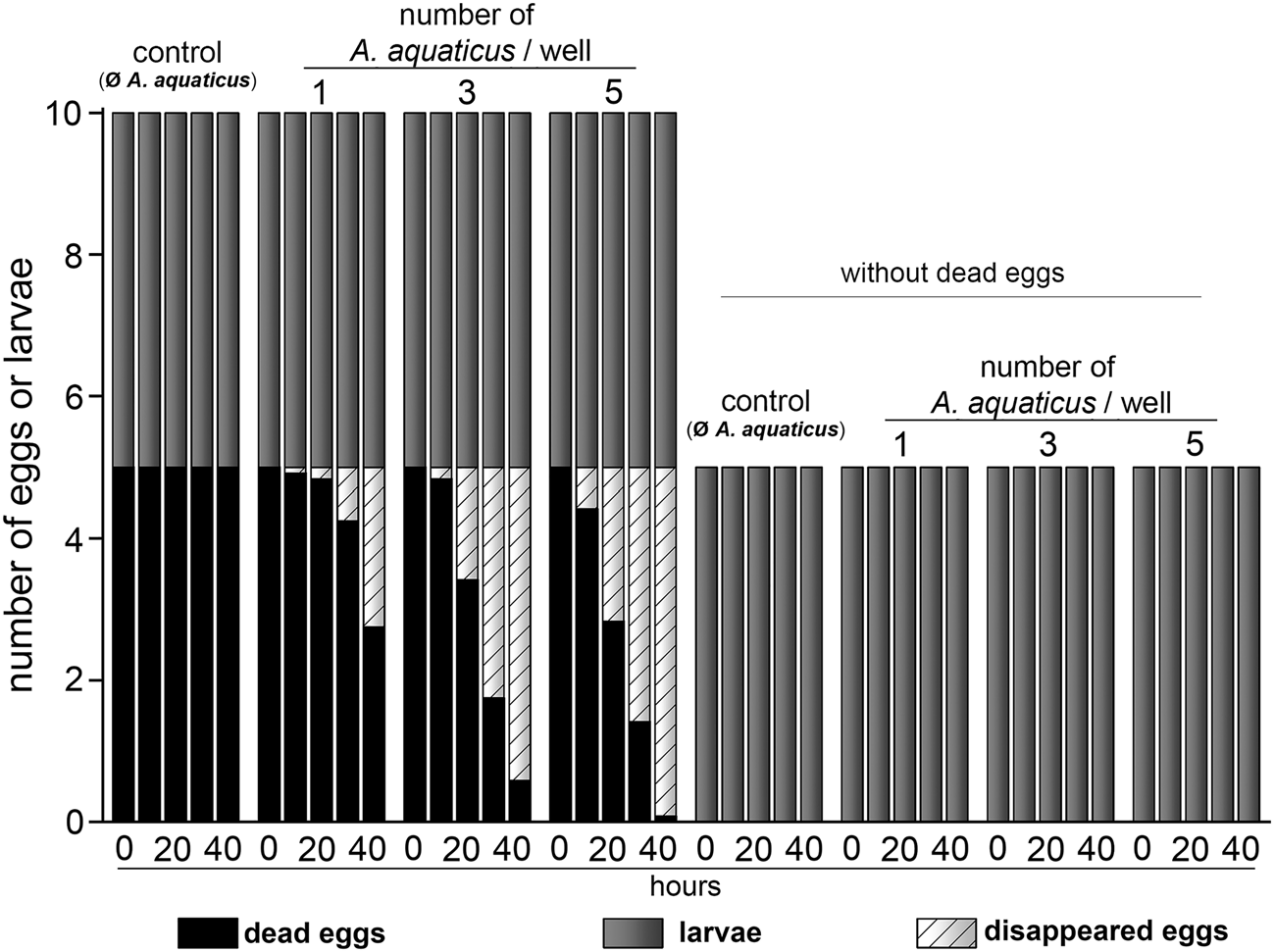
Effects of *A. aquaticus* on larval survival, with or without dead eggs as alternative feed source (E2). The stacked bar chart displays the number of dead eggs, disappeared eggs and hatched, live zebrafish larvae. Bar graphs indicate that the number of introduced hatched, live larvae remained unchanged during the 40-h incubation period, regardless of the presence or absence of dead eggs and/or *A. aquaticus*.

Similar to the previous experiment (E1), the number of dead eggs continuously decreased from the 5th hour in *A. aquaticus* containing wells. *A. aquaticus* did not harm the non-feeding larvae and there was no natural mortality detected, even when there was no alternative food source for the crustaceans. All introduced larvae survived throughout the experimental period, irrespective of the number of coexisting *A. aquaticus* specimens (Table 4, Fig. 2).

**Table 4:** Statistical analysis (MANOVA) of the effects of time, the number of *A. aquaticus* and their combination on egg batches during Experiment 2.

		df	Mean squares	F	p
Hours	UF	4	8.136	58.83	< 0.001
*A. aquaticus*	UF	3	6.298	45.54	< 0.001
Hours × *A. aquaticus*	UF	12	1.791	12.95	< 0.001
Error	UF	220	0.138		

*UF* unfertilised/dead eggs.

### Effects of *A. aquaticus* presence on larval vitality (E3)

**Figure 3:**
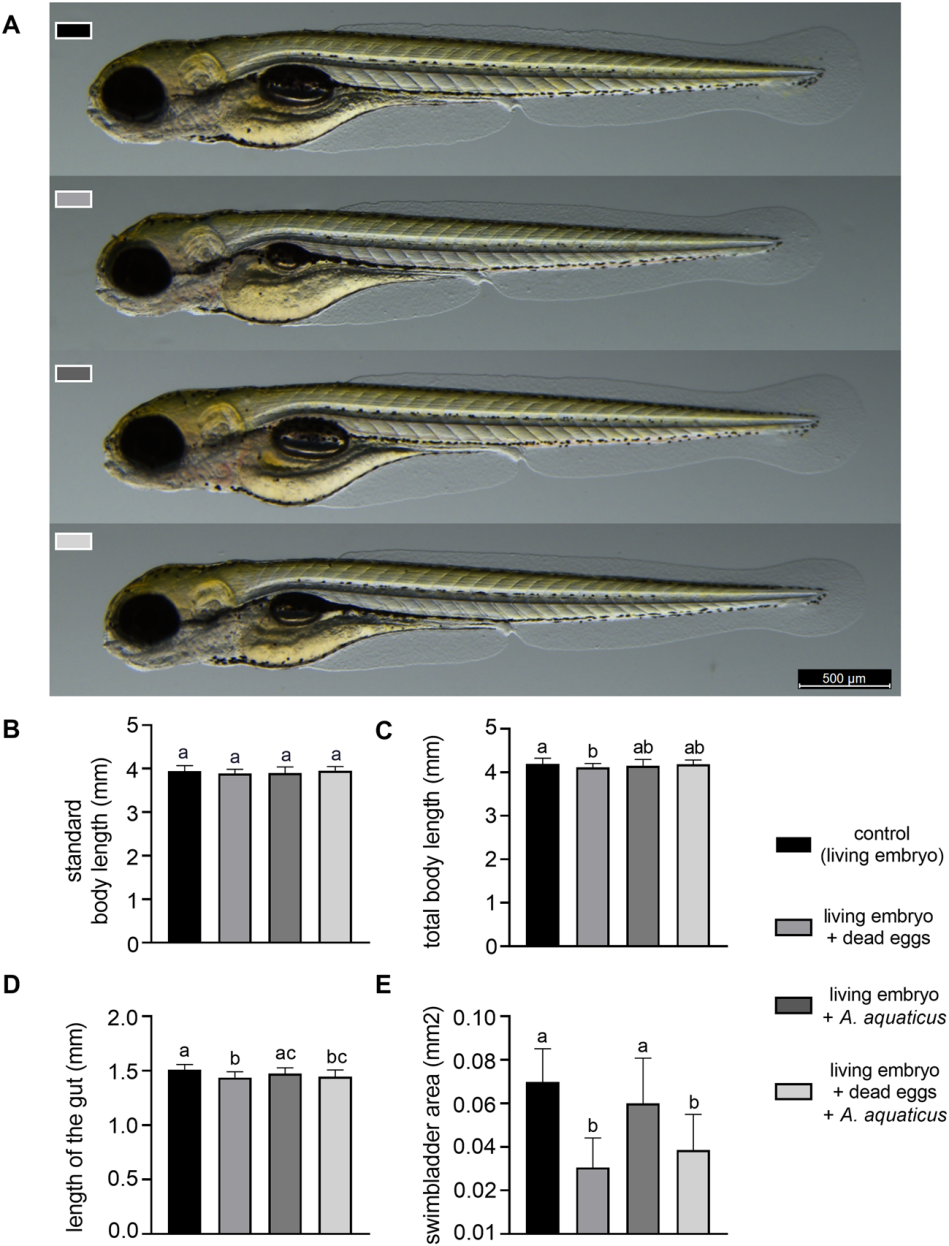
Effects of *A. aquaticus* presence during embryonic development on zebrafish larval morphology (E3). (**A**) Representative images of 120 hpf zebrafish larvae developed in the presence or absence of *A. aquaticus* and/or dead zebrafish eggs. Standard body length (**B**), total body length (**C**), length of the gut (**D**) and the swim bladder area (**E**) of 120 hpf zebrafish larvae developed in the presence or absence of *A. aquaticus* and/or dead zebrafish eggs. Data represent the mean and SD. Different letters indicate statistically significant differences, p < 0.05.

To assess the effects of *A. aquaticus* presence on larval vitality we determined the morphological and behavioral changes in the 120 hpf zebrafish larvae. There was no mortality during the experiments, although we did observe 10% whole body malformations in the co-presence of dead eggs and *A. aquaticus* during embryonic development. Regardless of this phenomenon, we did not detect any dramatic morphological alterations in the other larvae (Fig. 3A–E). No differences were found in the standard body length (Fig. 3B) however, interestingly, a slight but statistically significant decrease could be detected in the total body length and the gut length of the larvae developed in the presence of dead eggs compared to control (Fig. 3C,D). The gut length of the larvae developed in the presence of dead eggs also differed significantly from the group containing only *A. aquaticus* (Fig. 3D). Additionally, there was a more obvious and statistically significant decrease in the swim bladder area after incubation in the presence of dead eggs and in the co-presence of dead eggs and *A. aquaticus* as compared to the control and to the group containing only *A. aquaticus* (Fig. 3E).

**Figure 4:**
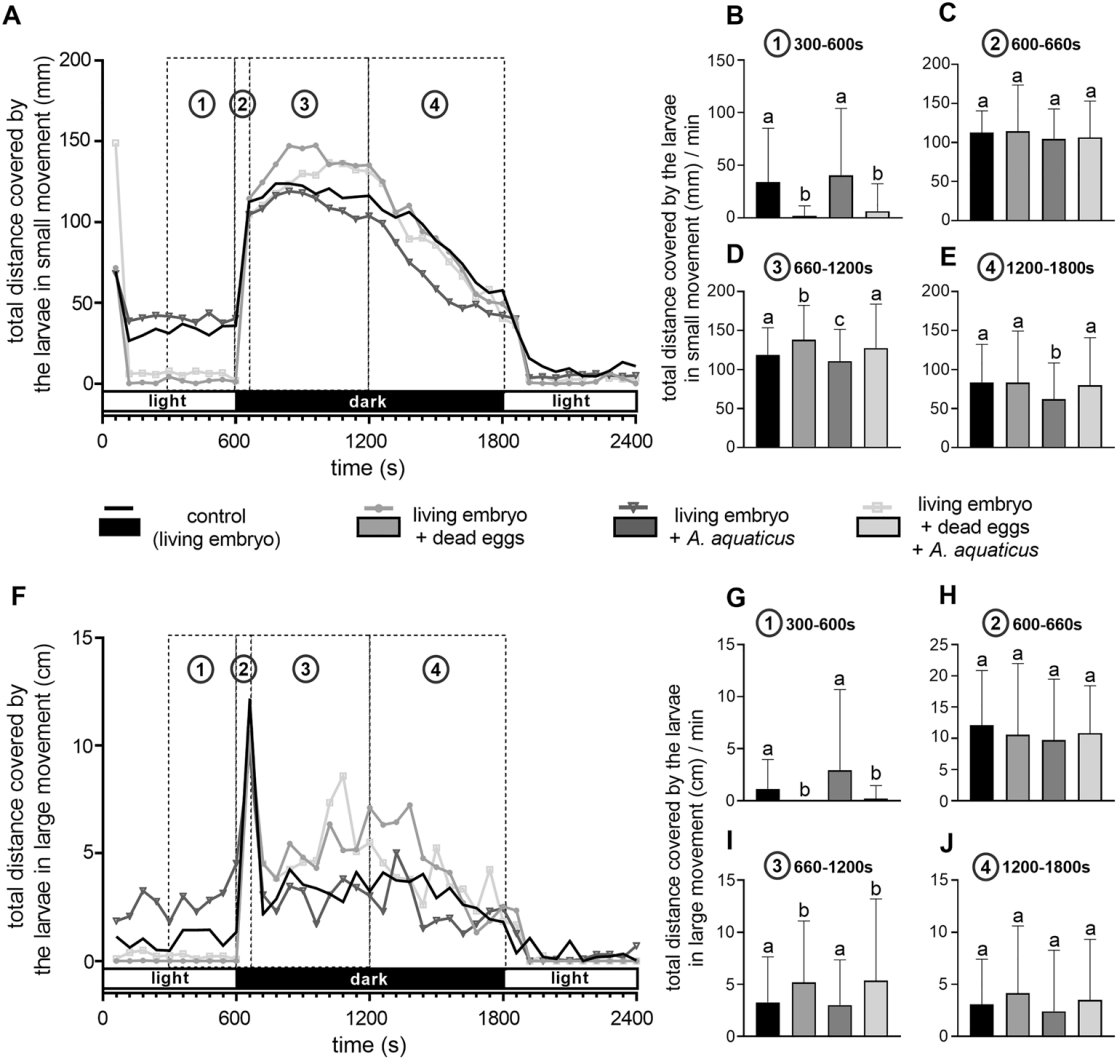
Effects of *A. aquaticus* presence during embryonic development on the locomotor activity of zebrafish larvae (E3). The figure summarizes the behavioral response of 120 hpf larvae developed in the presence or absence of *A. aquaticus* and/or dead zebrafish eggs during light–dark-light exposure. Under control conditions, the transition from light to dark elicits a sudden increase in the locomotor activity of zebrafish larvae. This activity decreases throughout the dark and the second light period. The whole test phase was divided into different periods to assess the potential alterations in the locomotor activity between the experimental groups. Locomotor activities were expressed as the distance traveled by the larvae with small or large movements. Line graphs showing the total distance covered by the larvae in small (**A**) and large (**F**) movements during a 10 min light—20 min dark—10 min light incubation with one minute resolution. Space between dashed lines (denoted as ‘1’, ‘2’, ‘3’, ‘4’) represents the statistically analyzed periods (300–600 s. (**B**,**G**) 600–660 s. (**C**,**H**) 660–1200 s. (**D**,**I**) 1200–1800 (**E**,**J**)) of the test. Data represent the mean and SD. Different letters indicate statistically significant differences, p < 0.05.

Next, we investigated the locomotor activity of the 120 hpf larvae performing the zebrafish behavioral assay. Locomotor activity measurement in zebrafish embryos and larvae is a frequently used model system for assessing the effects of environmental stressors. Normally, zebrafish larvae exhibit a sudden increase in locomotor activity following a transition from light to dark, followed by a decrease throughout the dark and the subsequent light period. These changes in the locomotor activity are regarded as characteristic behaviors of zebrafish at early larval stages^31,32^. Consequently, this allows for the comparison of potential differences in larval activity across various test periods. At the end of the incubation, we could not detect significant differences in the response of the larvae to the light–dark transition between the groups (Fig. 4A,C,F,H). However, the larvae which were developed in the presence of dead eggs or in the co-presence of dead eggs and *A. aquaticus* showed a significant decrease in locomotor activity during the pre-transition period (5 min before light–dark transition) (Fig. 4A,B,F,G), and a significant increase in the first half of the dark phase post transition compared to controls and to the group containing only *A. aquaticus* (Fig. 4A,D,F,I). Interestingly, the total distance covered by the larvae in small movements showed a slight, but significant decrease during the dark phase in the group containing only *A. aquaticus* as compared to every other group (Fig. 4A,D,E). However, this phenomenon was not observed for large movements (Fig. 4F,I,J).

Taken together, the presence of *A. aquaticus* during the embryonic development did not result in substantial morphological alterations, however, it slightly affected the locomotor activity of the larvae in a certain phase of a light–dark period. Moreover, dead eggs alone had a more remarkable impact on the measured morphological and behavioral parameters than the presence of *A. aquaticus*.

## Discussion

In the classical approach of biological control, predators and parasitoids are used which target the pathogens or pests themselves. The first attempts at chemical free control of oomycete parasites using two invertebrates, *Asellus militaris*, and *Gammarus pseudolimneaus*, were reported by Oseid^25^. According to their results, both invertebrates improved the survival rate of eggs by preventing fungal growth; however, *Gammarus* showed some predation on live eggs and larvae. Our knowledge is limited about the presence and significance of this phenomena in natural fish stocks.

The scraping feeding strategy of *A. aquaticus* makes it possible to ingest mycelia selectively from the surface of dead organic materials^28^. Bloor^33^ conducted feeding preference experiments with *A. aquaticus* and found that they prefer detritus which was partially digested by microbial communities and fungi. Possible reasons behind this experimental result could be that fungi can eliminate allelopathic chemicals of plants and through partial digestion they are able to make detritus more utilizable for the detrivores, which can also use the fungal enzyme system to degrade organic materials. Fungi also enrich the feed with their own micro- and macronutrient content^34^.

**Figure 5:**
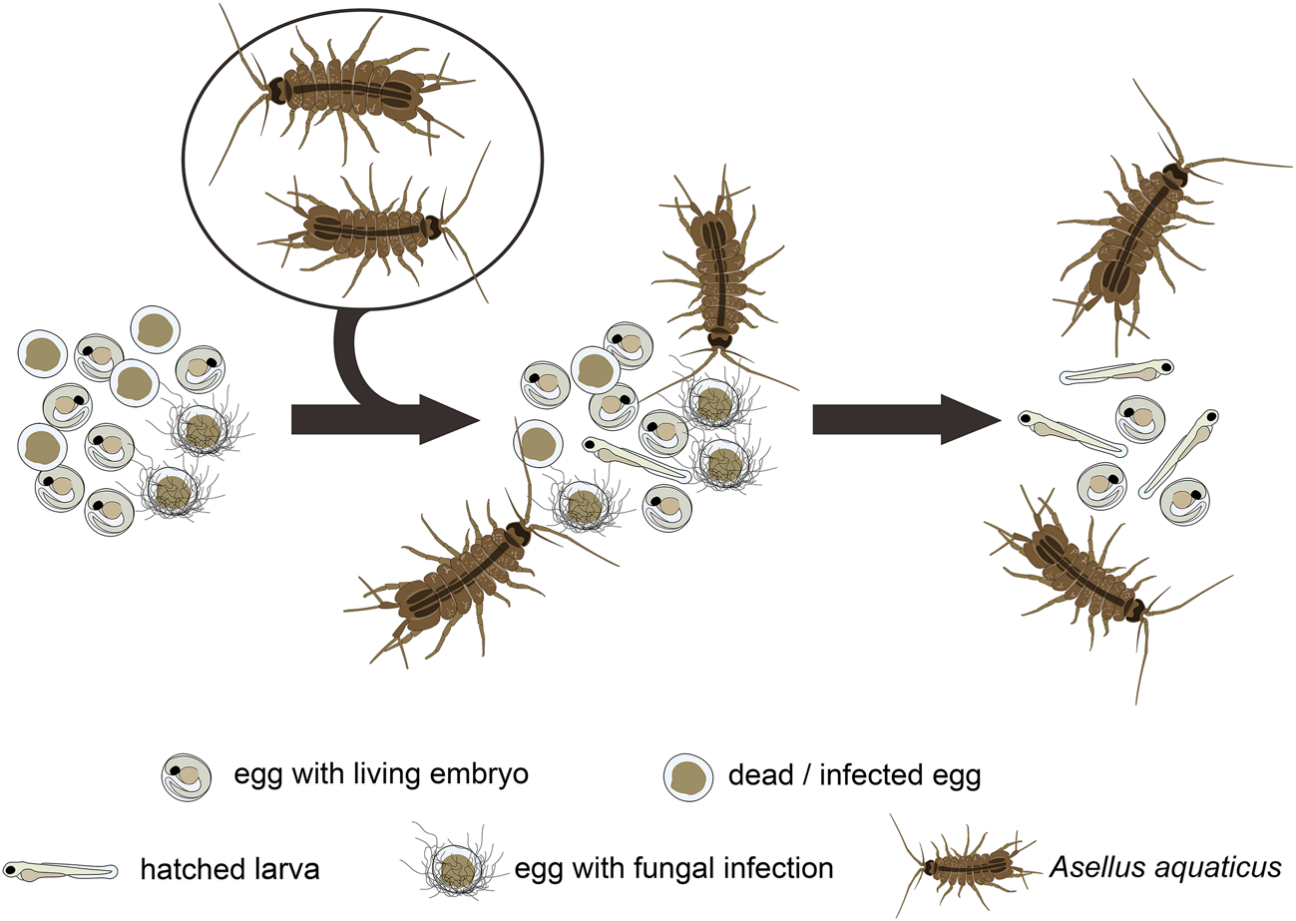
Schematic figure of the foraging features of *A. aquaticus* during fish embryo incubation. Dead and/or infected fish eggs can serve as a food source for *A. aquaticus.* These crustaceans have the ability to differentiate between dead and/or infected fish eggs and living embryos or larvae.

In this study, our theory on how *A. aquaticus* eliminates mycelia encompasses three main aspects: (a) there is direct grazing on the egg surfaces, aligning with their inherent feeding strategy; (b) we noted the consumption of infected and dead eggs; and (c) as a consequence of the movements and feeding behaviors of *A. aquaticus*, the eggshell of dead eggs can incur physical damage, potentially leading to “disappeared” eggs, as observed in this investigation. We believe that in this case the chorion undergoes damage, causing organic material to disperse into the water, which impedes the eggs from functioning as a substrate for fungal growth. The results of our study show that the feeding habits of *A. aquaticus* mitigate infection and prevent fertilized eggs from being entangled in fungal mycelium. (Fig. 5).

During our experiments none of the hatched larvae were harmed by *A. aquaticus*, even if no other feed source was provided. Other researchers found the same, that *A. aquaticus* specimens, as opposed to other crustaceans like *Gammaridae*, do not cause harm to young fish larvae^25,35^. However, it was also observed that hatching occurs faster in the presence of *A. aquaticus*. Most likely, the cause of this is the abrasion of egg shells due to the movements and feeding activity of the crustaceans. This phenomenon did not correlate with the number of *A. aquaticus* per well under our experimental conditions. This could be beneficial in a way, as shortened incubation periods also reduce the chance of fungal infection, though shorter incubation often results in smaller, weaker larvae, or limited hatching rate. Although we did not observe significant changes in the morphology of the larvae developed in the presence of *A. aquaticus*, the co-presence of dead eggs and *A. aquaticus* resulted in a low percentage (10%) of malformations. Besides, minor alterations were detected in the locomotor activity of the larvae in the presence of dead eggs and/or *A. aquaticus*. At the end of embryonic development, zebrafish larvae possess a complex and functional sensory system^36,37^. Consequently, the presence of *A. aquaticus* during the incubation period could affect the larvae's responses in behavioral tests conducted after incubation. It is important to note that most significant changes in morphology and locomotor activity were detected in groups where dead eggs were present. Detailed characterizations of the effects of dead eggs on the embryonic development of fish species are limited. Nevertheless, it can be hypothesized that the observed morphological and behavioral alterations may result from decaying organic matter. An elevated amount of organic matter can lead to hypoxia and release compounds that may affect the behavior and developmental rate of the embryos^38–40^. However, the exploration of the causal relationships related to these processes exceeds the scope of this study.

In summary, the presence of *A. aquaticus* during the zebrafish embryonic development had no considerable effects on larval vitality, at least until 120 hpf. However, it is possible that the greater density and larger size of *A. aquaticus* may have negative effects on the outcome of the incubation of relatively small, vulnerable fish eggs, which calls for further investigation.

Our present study demonstrates that *A. aquaticus* can reduce the number of dead zebrafish eggs. In addition, our preliminary experiments indicated that *A. aquaticus* was able to reduce the mass of water mold hyphae colonizing the eggshells of brown trout (*Salmo trutta fario*), a species with larger eggs (diameter of 5 mm) and longer embryonic development (44 days) as compared to zebrafish, and they were also able to consume the dead trout eggs (Supplementary Fig. 1). The elimination of dead eggs by *A. aquaticus* in large-scale operations can be limited. Hence, commercial hatcheries might need to administer multiple isopod inoculations to replace the manual egg picking method for disease management. A more feasible application of *A. aquaticus* could be with the semi-natural spawning of endangered fish species (e.g., *Misgurnus fossilis* and *Carassius Carassius*) or high value ornamental fish, where egg incubation and larvae rearing take place in the same unit, and the breeders do not show cleaning and fanning behavior. Control of pathogens by the elimination of the substrate for bacterial and fungal growth could improve the hatching rate and larval survival as well as general fish hygiene.

The applicability of this method in hatcheries should be tested in detail and the conditions of breeding and application of *A. aquaticus* should be optimized considering the effects of density, mating period, moulting and the different habits of the sexes and life stages^34,41^.

## Conclusions

According to the results of the current study, *A. aquaticus* can distinguish between living and dead fish eggs and are able to eliminate or reduce the number of dead eggs which can serve as a media for bacterial and fungal growth. We found that *A. aquaticus* do not harm eggs containing viable embryos and hatched larvae, even when alternative food sources were not available. We suggest that *A. aquaticus* can be a potential alternative biological control organism in special cases when small-scale egg incubation and larvae rearing are carried out at the same place (e.g., ornamental fish breeding or ex situ conservation). However, further investigations are required to determine the appropriate settings for utilizing this species in biological control, considering the specific fish species and environment involved.

### Supplementary Information

Supplementary Tables.

Supplementary Figure 1.

## Data Availability

All data generated or analysed during this study are included in this published article (and its Supplementary Information files).
