# Supplementary figures and images for: Removal of dead fish eggs by Asellus aquaticus as a potential biological control in aquaculture
Source: Sci Rep. 2024 Mar 27;14:7295. doi: 10.1038/s41598-024-57903-4 (PMC10973432; doi:10.1038/s41598-024-57903-4)

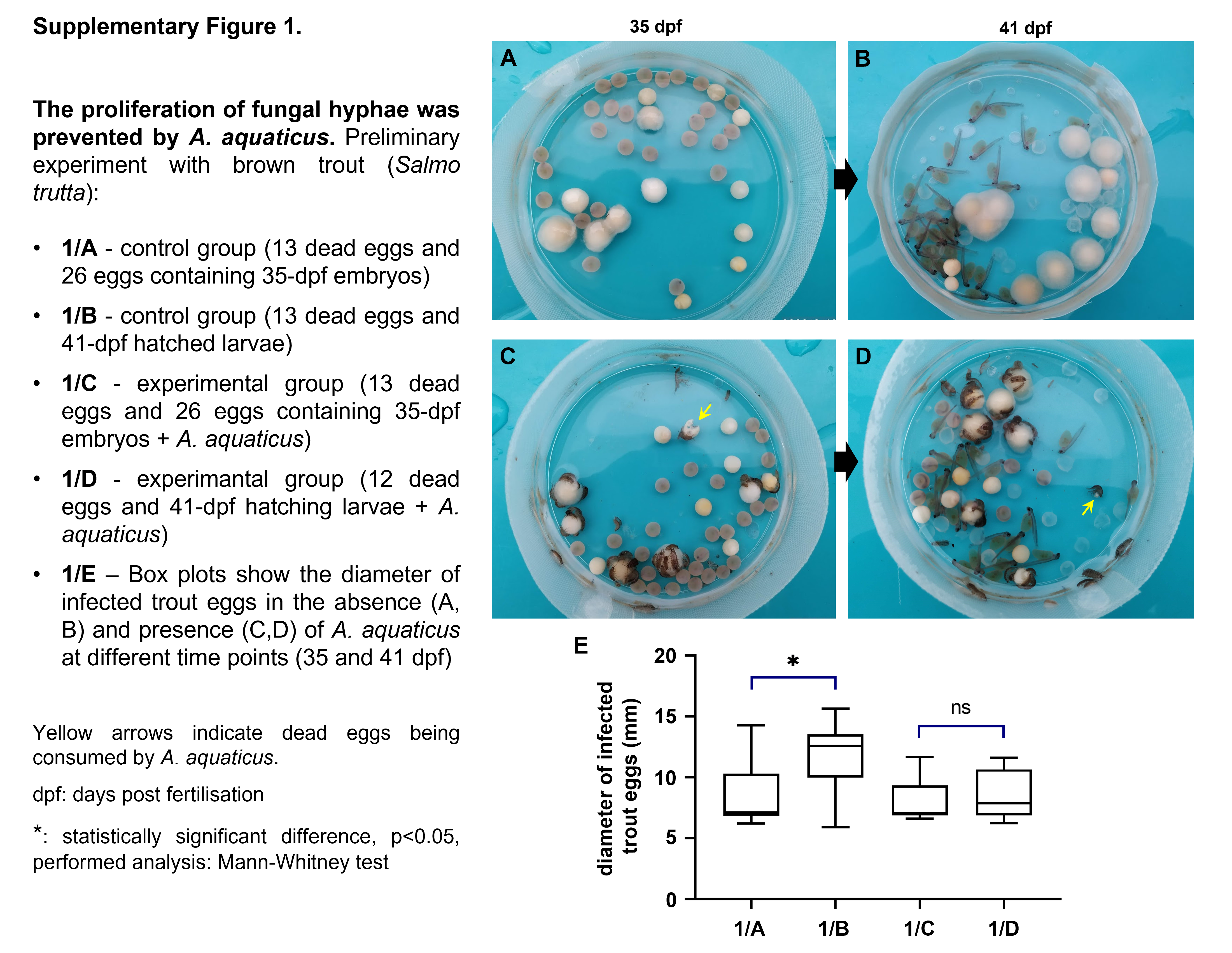

Supplement: Supplementary file 2 — Supplementary Figure 1. [file 41598_2024_57903_MOESM2_ESM.tif]
